# Health impact of foods: Time to switch to a 3D-vision

**DOI:** 10.3389/fnut.2022.966310

**Published:** 2022-07-18

**Authors:** Mathilde Touvier, Bernard Srour, Serge Hercberg, Pilar Galan, Emmanuelle Kesse-Guyot, Chantal Julia

**Affiliations:** ^1^Sorbonne Paris Nord University, Inserm U1153, Inrae U1125, Cnam, Nutritional Epidemiology Research Team (EREN), Epidemiology and Statistics Research Center–Université Paris Cité (CRESS), Bobigny, France; ^2^Public Health Department, Avicenne Hospital, AP-HP, Bobigny, France

**Keywords:** nutrition, food processing, environmental contaminants, prevention, chronic disease risk, front-of-package labeling, Nutri-Score

The health impact of the “nutritional” dimension of foods (i.e., the amounts of sugar, saturated fats, salt, energy, fiber, protein, minerals, vitamins, etc.) is well established (1). Indeed, based on thousands of epidemiological and experimental studies globally, high levels of evidence have been reached regarding the deleterious impact of an excessive consumption of foods rich in salt, sugar, saturated fats and a limited consumption of foods high in fiber, vitamins and minerals on the risk of several cancers, obesity, cardiovascular diseases, diabetes, and mortality. Hence, national official food-based dietary guidelines have been issued and are overall consistent across countries. Additionally, nutrients of public health concern serve as a basis for the large majority of the currently available front-of-package labeling systems. Indeed, numerous national and international experts' committees (including the World Health Organization) recommend to display an interpretive nutrition labeling system on front-of-pack of foods aiming to help consumers understand, at a glance, the nutritional quality of a food product at the time of purchase, ultimately enabling consumers to choose between comparable food products. Amongst these labels, Nutri-Score, a gradual 5-letter/5-color front-of-pack nutrition label already adopted in 7 European countries (France, Belgium, Germany, Spain, the Netherlands, Luxembourg and Switzerland), aims to guide consumers toward nutritionally healthier food choices and incentivize food manufacturers to improve the nutritional quality of their recipes. Several studies conducted in large prospective cohort studies in France, Spain and in the European EPIC cohort (carried out in 10 European countries) found associations between the Food Standards Agency nutrient profiling system, which serves as the Nutri-Score's algorithm, with the risk of chronic diseases (cancers, cardiovascular diseases, weight gain, metabolic syndrome, etc.) and mortality (2). Furthermore, Nutri-Score has shown good performance in studies investigating its perception and understanding, as well as its actual impact on food choices, including in low-income populations (3). Nutri-Score has also a crucial role to play in encouraging food companies to improve the nutritional composition of their products.

Beyond this nutritional dimension, the past 5 years have witnessed a strong dynamism of research which today leads to widen this vision of the health impact of foods, by integrating an additional key dimension: (ultra)processing/formulation (4). Indeed, >50 recent prospective studies have shown links between the consumption of so-called “ultra-processed foods” according to the NOVA classification (i.e., having undergone major processing and/or containing food additives or other industrial substances such as hydrogenated oils, maltodextrin, glucose syrup, etc.) and an increased risk of many non-communicable diseases (5). These studies were conducted in various populations worldwide (e.g., Sun and Predimed cohorts in Spain, NutriNet-Santé in France, Nurses Health Study in the USA, UK Biobank), and were adjusted for several components (sugar, salt, saturated fatty acids and energy) of the nutritional quality of the diet. Experimental studies highlighted health effects of various non-nutritional components conveyed by these foods, such as certain additives or contaminants formed during processing. However, information on “ultra-processed” products *per se*, enabling consumers to identify them, has not yet been directly transposed at the level of food packaging.

On the other hand, several studies (particularly in the French NutriNet-Santé cohort) observed a lower risk of chronic diseases among the highest consumers of organic foods or those less exposed to pesticide residues (6). There is already in Europe an information label available on the packs of foods, the European Union organic label, corresponding to a quality label certifying that a product complies with the European Union Regulation on organic agriculture, based on the ban on synthetic fertilizers and pesticides.

Consequently, with regards to current knowledge about the 3 aspects stated above, these 3 different dimensions are all linked to health outcomes, and need to be all considered to obtain a more complete picture of the overall health impact of foods. None is exclusive and able to summarize, by itself, how the food product may impact health. Here is a practical example: some chips found in supermarkets may not be “ultra-processed,” but they present a limited nutritional quality, with high amounts of salt, fat and energy. An organic cookie generally contains less pesticide residues than its conventional equivalent, but it may be ultra-processed, and its nutritional quality is not necessarily better. Finally, a diet soda does not have a bad nutritional quality (none-to-low calories and sugar), but it is typically ultra-processed (containing artificial sweeteners, dyes, etc.).

These three dimensions can certainly be inter-related (e.g., “ultra-processed” foods on average do have a lower nutritional quality), but they are not collinear and correspond to complementary concepts.

The issue is that messages are currently circulating among scientists, physicians, and the lay public, suggesting that one (or the other) of these dimensions would be sufficient to ”summarize“ the other two, and to convey a global picture on how healthy a food product is. This partial view is reductionist and misleading. Some claim that the fact that a food is not ultra-processed would be a guarantee of a favorable nutritional quality, which is obviously refuted by the example of industrial chips above. Likewise, the “halo” effect is often used by manufacturers as a marketing argument to give an overall healthy image to a fatty/sweet, but organic product, while this “organic” label does not provide direct information on the remaining two health dimensions of the product (i.e., nutritional quality, and level of processing/formulation).

For consumers, these intertwined concepts may seem confusing because they require to make a trade-off on which dimension(s) to favor. While some questions remain unanswered, considering the current state of knowledge, it is important to ensure that consumers have access to an adequate information to evaluate the quality of a food product, within each of these 3 dimensions, in order to make globally healthier choices. Dietary guidelines could therefore recommend: a) choosing (within comparable products) foods with a better nutritional quality - i.e., having a better Nutri-Score, b) preferring non-to-minimally processed foods rather than ultra-processed foods, and c) favoring organic foods as much as possible (especially for plant foods/ingredients) when an organic alternative is accessible.

Practically, developing adapted labels to cover these food dimensions, supported by mass communication campaigns can be effective tools to ensure better food choices. In terms of front-of-pack labeling, these 3 health dimensions of foods could be translated by a) the Nutri-Score, providing information on the nutritional dimension, b) an additional graphic mention (e.g., black band surrounding the Nutri-Score) specifying whether the food is “ultra-processed” (based for instance on an operational transposition of the NOVA-4 category), as the strongest evidence for associations between food processing and chronic diseases was specifically reported for this category, and c) the “organic” logo, providing information on the contaminant/pesticide dimension (see Figure 1). It is obviously crucial to support these graphical tools with massive communication campaigns to educate the consumers on each of these dimensions, and provide an adapted and accessible “user guide” for these labels.

**Figure 1:**
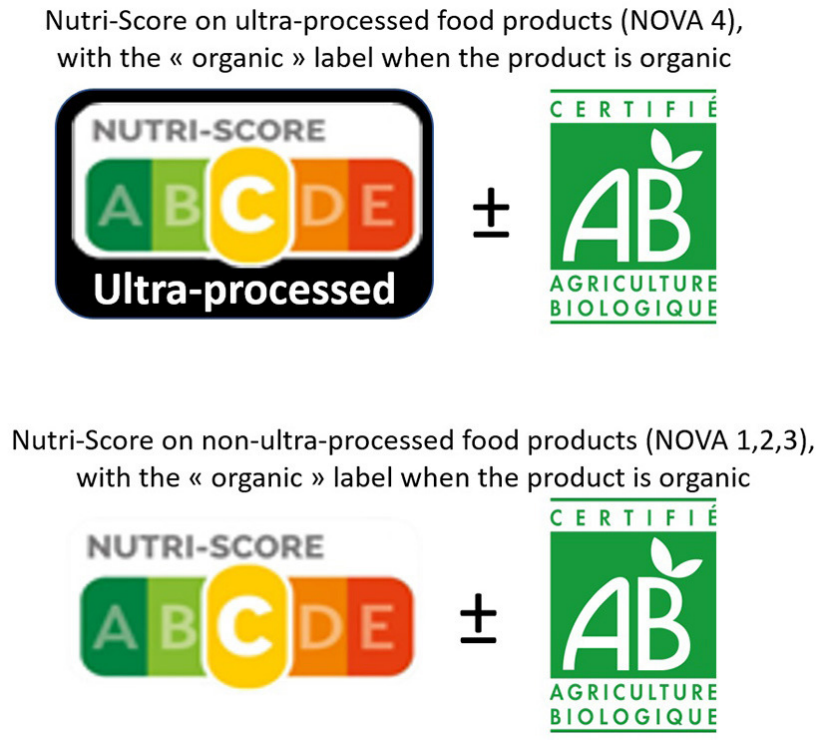
Proposed front-of-package label, including the 3 health dimensions of foods.

Finally, it is important to keep in mind that science is a dynamic process. In the next decade, the research work in progress will lead to better characterize the health impacts of nutritional compounds, pesticides, additives, and contaminants from industrial processes, including knowledge on mixture/cocktail effects. This will permit to optimize regulations, labeling, and recommendations based on this more complete picture, in a constant perspective of patients and citizens' health preservation. Of course, beyond these 3 health-related dimensions, other aspects must be considered such as planetary or socio-economic impacts linked to production modes.

Despite the fact that current scientific knowledge does not allow to prioritize health risks or benefits associated with each dimension, we know today that they are all important to consider. While developing research programs to obtain further scientific answers, we can (and must) already act to provide consumers with the adequate information and tools about these 3 dimensions. As wisely said by Sir Austin Bradford Hill: “*All scientific work is incomplete—whether it be observational or experimental. All scientific work is liable to be upset or modified by advancing knowledge. That does not confer upon us a freedom to ignore the knowledge we already have, or to postpone the action it appears to demand at a given time.”*

Thus, when it comes to the health impact of foods, it is now time to switch to a 3D-vision.

## Author contributions

All authors listed have made a substantial, direct, and intellectual contribution to the work and approved it for publication.

## Conflict of interest

The authors declare that the research was conducted in the absence of any commercial or financial relationships that could be construed as a potential conflict of interest.

## Publisher's note

All claims expressed in this article are solely those of the authors and do not necessarily represent those of their affiliated organizations, or those of the publisher, the editors and the reviewers. Any product that may be evaluated in this article, or claim that may be made by its manufacturer, is not guaranteed or endorsed by the publisher.
